# Providing care in underresourced areas: contribution of the physician assistant/associate workforce

**DOI:** 10.1186/s12913-024-11190-x

**Published:** 2024-07-25

**Authors:** Mirela Bruza-Augatis, Bettie Coplan, Kasey Puckett, Andrzej Kozikowski

**Affiliations:** 1 National Commission on Certification of PAs, Johns Creek, GA USA; 2 Department of Physician Assistant Studies, Northern Arizona University, Phoenix, AZ USA

**Keywords:** Underresourced areas, Medically underserved areas, Health professional shortage areas, Healthcare workforce, Physician assistants

## Abstract

**Background:**

Prior studies suggest that physician assistants/associates (PAs) are more likely than physicians to work in underresourced areas. However, data characterizing the current PA workforce in health professional shortage areas (HPSAs) and medically underserved areas (MUAs) are lacking.

**Methods:**

We analyzed the 2022 cross-sectional dataset from a comprehensive national database to examine the demographic and practice characteristics of PAs working in HPSAs/MUAs compared to those in other settings. Analyses included descriptive and bivariate statistics, along with multivariate logistic regression.

**Results:**

Nearly 23% of PAs reported practicing in HPSAs/MUAs. Among PAs in HPSAs/MUAs, over a third (34.6%) work in primary care settings, 33.3% identify as men, 15.6% reside in rural/isolated areas, and 14.0% are from an underrepresented in medicine (URiM) background. Factors associated with higher odds of practicing in a HPSA/MUA included residing in rural/isolated settings, URiM background, and speaking a language other than English with patients.

**Conclusions:**

As the PA profession grows, knowledge of these attributes may help inform efforts to expand PA workforce contributions to address provider shortages.

**Supplementary Information:**

The online version contains supplementary material available at 10.1186/s12913-024-11190-x.

## Introduction

Since the 1960s, a scarcity of healthcare providers in rural and other underresourced settings in the United States (US) has challenged healthcare policymakers [1]. Despite various efforts to increase the provision of services in shortage areas, a maldistribution of healthcare providers persists [2, 3]. Difficulty retaining providers in underresourced areas compounds the issue [4].

As defined by the Health Resources and Services Administration (HRSA), two main types of shortage areas exist in the US: health professional shortage areas (HPSAs) and medically underserved areas (MUAs) [5]. HPSAs are geographic areas, populations, or facilities with a shortage of primary care physicians, dentists or mental health providers [6]. The threshold for primary care HPSA designation is a population-to-provider ratio of 3,500:1 compared to an average in the US of 1,320:1 [7, 8]. MUAs are geographic areas that lack access to primary care services. The number of primary care physicians per 1,000 population and percent of population at the federal poverty level are among the criteria for MUA [6]. The majority of HPSA and MUA designations exist in rural areas, defined as having populations of less than 50,000 people [5, 9]. However, shortage areas in more urban areas also encompass large numbers of people. At the end of 2023, more than 100 million people in 8,544 primary medical HPSA designations had access to 47.6% fewer physicians than those in non-HPSA areas [5, 8]. Among them, nearly half resided in non-rural HPSAs [8]. As of March 2024, 15 million people lived in 3,442 MUA designations [5].

Physician shortages in underresourced rural areas are projected to worsen [2, 10], and the proportion of healthcare visits managed by physician assistants/physician associates (PAs) and nurse practitioners (NPs), collectively known as advanced practice providers (APPs), appears to be increasing across various settings [11, 12]. Thus, the rapidly expanding numbers of APPs may be increasingly called on to provide care to HPSA and MUA patient populations [13–15]. Physicians launched the PA profession in the mid-1960s mainly to address a growing lack of access to medical care [16]. As of December 31, 2022, 23.0% of the 168,318 board certified PAs were in primary care (family medicine, general internal medicine, and general pediatrics) [17]. In addition, PAs practicing in rural and other underresourced settings are more likely to provide primary care services than PAs in urban and adequately resourced areas [18, 19]. Yet very little is known about the characteristics of this subset of the workforce.

Muma and colleagues conducted a national survey of 2,048 PAs in 2008, which showed that similar to findings among physicians, PAs from racial and ethnic minority groups were more likely than those from non-minority groups to practice in primary care and care for underserved patients – 38.8% compared to 29.3% practiced in primary care and 31.9% compared to 19.3% provided care to underserved patients [20]. This study also found that PAs who were 39 years of age or older and from low-income families were also more likely to practice in these settings [20]. Another national study examining PA students found that those from minority groups and from rural areas or less affluent backgrounds expressed greater interest in practicing in a MUA [21].

Additional national data on the demographic characteristics of PAs likely to practice in HPSA/MUA settings are lacking. Regarding clinical activity, prior studies show that PAs in rural and underresourced areas tend to have a broader scope of practice than those in metropolitan areas and that APPs manage a higher proportion of patient visits in nonmetropolitan compared to metropolitan locations [14, 18]. In fact, PAs working in rural regions are more likely to provide care in underresourced settings and federally qualified health center (FQHC) designations than PAs working in urban areas [22, 23]. However, much of the research on PAs in rural and underresourced areas is over 10 years old. Furthermore, potential differences between PA experiences in HPSAs/MUAs compared to non-underresourced settings have not been fully explored.

To inform efforts to recruit and retain PAs likely to practice in underresourced areas and optimize their contributions, a current and more thorough understanding of PAs who work in HPSAs/MUAs is needed. To address this need, we analyzed data from the National Commission on Certification of Physician Assistants (NCCPA), the sole organization responsible for national board certification of PAs in the US, to gain a comprehensive picture of the characteristics of PAs and PA practice patterns in HPSA/MUA settings. Because all states require initial NCCPA certification for licensure, and many states and employers require maintenance of certification, most US PAs regularly interface with the NCCPA and respond to queries [24]. Consequently, while many prior survey studies focusing on PAs in underresourced areas are limited by low response rates or small sample sizes, NCCPA data is highly representative of the US PA population due to its higher response rate (83.7% in 2022) [17].

## Data source and methods

NCCPA has collected data on demographic and practice characteristics of nationally board certified PAs through an online interface titled the ‘PA Professional Profile’ since 2012. PAs can update their profile characteristics at any time and are prompted to do so when they log in to the NCCPA website to complete certification requirements. For example, to maintain certification, PAs must log in to the NCCPA website a minimum of once every two years to document the completion of continuing medical education (CME) credits. The PA Profile is regularly updated, with questions periodically added or amended. In 2021, a question asking respondents whether they provide care to patients in designated HPSA/MUA areas was added; response choices included ‘yes,’ ‘no,’ and ‘not sure.’ The definition of HPSA/MUA settings as defined by HRSA was provided with the question.

For this cross-sectional study, we examined 2022 PA Profile data and included responses from PAs who had confirmed or updated their profile within the last three years (*n* = 140,815). We conducted descriptive and bivariate analyses among PAs working in HPSAs/MUAs and those not working in HPSAs/MUAs by comparing demographic factors (e.g., age at initial certification, gender, race, ethnicity, urban/rural residence, US region or residence, highest degree completed, number of years certified), practice characteristics, job satisfaction, burnout, and intention to leave the clinical position in the next year.

Job satisfaction was measured on a seven-point scale from ‘Completely Dissatisfied’ to ‘Completely Satisfied’ and then categorized into ‘Not Satisfied’ and ‘Satisfied.’ Burnout was measured on a five-point scale, then dichotomized into ‘No Symptoms of Burnout’ and ‘One or More Symptoms of Burnout’ [25, 26] Practice characteristics examined included PA specialty, clinic type (e.g., hospital, office-based private practice), information related to clinic vacancies, types of services provided, and payer mix. Home residence is administrative data needed for correspondence between NCCPA and PAs, and thus provides the most complete and accurate data on respondent location. It was, therefore, used to determine PA’s geographic location. On the other hand, practice/work addresses are self-reported and include considerable missing data (44.8%). The PA Profile lists the residence geographic area as urban, large rural, small rural, and isolated, utilizing the definition of Rural-Urban Community Areas (RUCA) codes [27].

In addition, we performed a multivariate logistic regression to determine which demographic characteristics of PAs were associated with working in HPSAs/MUAs. The outcome variable – whether or not respondents reported they practice in a HPSA/MUA setting (*n* = 106,253) was dichotomized as ‘yes’ and ‘no/not sure.’ The ‘not sure’ responses, which were an option on the PA Profile for participants, comprised 19.7% (*n* = 27,774) of the sample. Consequently, we conducted a sensitivity analysis to determine if there were differences in the results of including or excluding the ‘not sure’ responses. Since no differences were found, we reported the results with ‘not sure’ to preserve the study’s statistical power. All analyses were conducted using SPSS Statistical Software (Version 29.0; IBM Corp Armonk, NY, USA). The Sterling Institutional Review Board (IRB#9942) considered this study exempt.

## Results

### Demographic characteristics

By the end of 2022, 140,815 of the 168,318 nationally board certified PAs had completed or updated their PA Profile responses within the last three years. Of the PAs who updated their profile, 118,495 reported working in at least one clinical position. Among PAs who practice clinically, 106,253 responded to the question asking whether they provide care to patients in HPSA/MUA designations – resulting in an 89.7% response rate. Among the PAs who responded to the HPSA/MUA question, 22.8% (*n* = 24,180) reported providing care in a HPSA/MUA setting.

Bivariate analyses revealed that, compared to PAs who reported not working in a HPSA/MUA (*n* = 82,073), those working in HPSAs/MUAs (all *p* < 0.001) were more likely to identify as men (33.3% versus 29.4%), Black/African American (4.8% versus 3.0%), and Hispanic/Latino(a/x) (9.5% versus 6.0%), respectively (Table 1). An examination of the race and ethnicity breakdown by practice setting showed that PAs from groups underrepresented in medicine (URiM) [28] comprised 14.0% (*n* = 3,299) of those working in HPSAs/MUAs and 9.0% (*n* = 7,005) of those working in other settings (*p* < 0.001).

**Table 1:** Demographic characteristics of PAs working in HPSA/MUA settings vs. PAs not working in HPSA/MUA settings (*N* = 106,253)

	PAs working in HPSA/MUA settings (*N* = 24,180)	PAs not working in HPSA/MUA settings^a^ (*N* = 82,073)	*P*-value^b^
Age at Initial Certification			
Mean (SD)	29.9 (6.1)	28.8 (5.4)	< 0.001
Median (IQR)	28 (26–32)	27 (25–31)	
Gender^c^			
Female	16,121 (66.7%)	57,939 (70.6%)	< 0.001
Male	8,057 (33.3%)	24,124 (29.4%)	
Race			
White	18,974 (82.4%)	67,282 (85.4%)	< 0.001
Asian	1,248 (5.4%)	5,079 (6.4%)	
Black/African American	1,114 (4.8%)	2,329 (3.0%)	
American Indian or Alaska Native	163 (0.7%)	202 (0.3%)	
Native Hawaiian/Pacific Islander	70 (0.3%)	233 (0.3%)	
Multi-race	622 (2.7%)	1,687 (2.1%)	
Other	849 (3.7%)	1,986 (2.5%)	
Ethnicity			
Non-Hispanic/Non-Latino(a/x)	21,006 (90.5%)	74,424 (94.0%)	< 0.001
Hispanic/Latino(a/x)	2,199 (9.5%)	4,725 (6.0%)	
Underrepresented in Medicine (URiM) Status^d^			
Non-URiM	19,533 (85.6%)	71,175 (91.0%)	< 0.001
URiM	3,299 (14.0%)	7,005 (9.0%)	
US Region (Residence)			
Northeast	5,401 (22.4%)	20,522 (25.1%)	< 0.001
Midwest	4,557 (18.9%)	16,415 (20.1%)	
South	7,673 (31.9%)	28,937 (35.4%)	
West	6,447 (26.8%)	15,905 (19.4%)	
Urban/Rural Setting (Residence)^e^			
Urban	20,276 (84.4%)	77,441 (94.9%)	< 0.001
Rural/Isolated	3,749 (15.6%)	4,177 (5.1%)	
Speaks a Language Other Than English			
No	16,670 (70.9%)	63,709 (79.7%)	< 0.001
Yes	6,857 (29.1%)	16,210 (20.3%)	
Highest Degree Completed			
Certificate Program	256 (1.1%)	720 (0.9%)	< 0.001
Associate’s Degree	252 (1.0%)	616 (0.8%)	
Bachelor’s Degree	3,556 (14.7%)	11,991 (14.6%)	
Master’s Degree	19,192 (79.4%)	66,545 (81.1%)	
Doctorate Degree	744 (3.1%)	1,701 (2.1%)	
Other	163 (0.7%)	472 (0.6%)	
Completed PA Postgraduate Fellowship/Residency			
No	22,764 (94.2%)	77,615 (94.7%)	< 0.001
Yes	1,395 (5.8%)	4,364 (5.3%)	
Years Certified as a PA			
Less than 10	13,018 (53.8%)	41,253 (50.3%)	< 0.001
11 to 20	7,082 (29.3%)	25,375 (30.9%)	
21 and over	4,080 (16.9%)	15,445 (18.8%)	
^a^PAs not working in HPSA/MUA setting include responses for ‘no’ and ‘not sure.’ ^b^Pearson Chi-Square analysis, Independent-Samples Mann-Whitney U Test when appropriate. ^c^Gender is part of the NCCPA administrative database obtained by PA programs and is reported as male, female, non-binary, and prefer not to answer. The non-binary and prefer not to answer responses include less than 0.1% and are not included in this analysis. ^d^URiM was defined based on the AAMC definition (AAMC, 2004). Based on current US population demographics, PAs who identified as white, Asian, and non-Hispanic/Latino(a/x) [plus other or multi-race] were included in the non-URiM group, whereas PAs who identified as Black/African American, American Indian or Alaska Native, Native Hawaiian/Pacific Islander, and Hispanic/Latino(a/x) [plus other or multi-race] were included in the URiM group. ^e^Urban-rural setting includes large rural, small rural, and isolated geographical US areas using Rural-Urban Community Area (RUCA) Codes (source: https://www.ers.usda.gov/data-products/rural-urban-commuting-area-codes/) Abbreviations: SD = Standard Deviation; IQR = Interquartile Range Source: 2022 National Commission on Certification of Physician Assistants (NCCPA) PA Professional Profile			

Table 1 also shows additional demographic attributes of PAs providing care in HPSA/MUA settings. Compared to their counterparts, a higher proportion of PAs in HPSAs/MUAs were certified for ten years or less (53.8% versus 50.3%), reported speaking a language other than English with their patients (29.1% versus 20.3%), completed a PA postgraduate fellowship/residency program (5.8% versus 5.3%), and held a doctorate degree (3.1% versus 2.1%), respectively (all *p* < 0.001).

### Specialty, practice, and clinic characteristics

An examination of medical specialties showed that PAs working in HPSAs/MUAs versus their counterparts were more likely to practice in primary care (34.6% versus 19.7%), emergency medicine (13.6% versus 10.2%), hospital medicine (4.0% versus 3.5%), psychiatry (2.9% versus 2.0%), obstetrics/gynecology (1.3% versus 1.1%), and addiction medicine (0.9% vs. 0.4%; Table 2) (all *p* < 0.001). In addition to comparisons by specialty, Table 2 shows comparisons of the characteristics of the clinics in which PAs are working, including information on clinic vacancies. Overall, PAs working in HPSAs/MUAs were more likely to provide care in the types of clinics associated with HPSA/MUA designations, such as rural health clinics, community health clinics, and FQHCs. Furthermore, compared to PAs working in all other settings, PAs rendering care in HPSA/MUA designations were also more likely to report that their place of employment was currently recruiting/hiring PAs (43.3% versus 35.3%; *p* < 0.001), and PA positions at their place of employment remained unfilled for six or more months (28.2% versus 20.2%; *p* < 0.001).

**Table 2:** Practice and other important characteristics of PAs working in HPSA/MUA settings vs. PAs not working in HPSA/MUA settings (*N* = 106,253)

	PAs working in HPSA/MUA settings (*N* = 24,180)	PAs not working in HPSA/MUA settings^a^ (*N* = 82,073)	*P*-value^b^
Specialty			
Primary Care^c^	8,338 (34.6%)	15,892 (19.7%)	< 0.001
Emergency Medicine	3,296 (13.6%)	8,270 (10.2%)	
Surgery-Subspecialties	3,122 (13.0%)	16,539 (20.5%)	
Internal Medicine-Subspecialties	1,913 (7.9%)	8,480 (10.5%)	
Hospital Medicine	960 (4.0%)	2,837 (3.5%)	
Psychiatry	690 (2.9%)	1,651 (2.0%)	
Surgery-General	606 (2.5%)	2,546 (3.1%)	
Dermatology	524 (2.2%)	3,909 (4.8%)	
Critical Care Medicine	421 (1.7%)	1,644 (2.0%)	
Obstetrics/Gynecology	323 (1.3%)	926 (1.1%)	
Pediatrics – Subspecialties	309 (1.3%)	1,027 (1.3%)	
Addiction Medicine	225 (0.9%)	341 (0.4%)	
Other	3,383 (14.0%)	16,772 (20.7%)	
Primary Practice Clinic Type			
Hospital	9,854 (40.9%)	33,585 (41.5%)	< 0.001
Office-Based Private Practice	6,559 (27.2%)	32,782 (40.6%)	
Community Health Center (FQHC)^d^	2,563 (10.6%)	458 (0.6%)	
Rural Health Clinic	1,220 (5.1%)	271 (0.3%)	
Urgent Care	1,075 (4.5%)	4,720 (5.8%)	
Federal Government	1,056 (4.4%)	3,898 (4.8%)	
Public or Community Health Clinic (non-FQHC)	277 (1.2%)	439 (0.5%)	
Behavioral/Mental Health Facility	250 (1.0%)	438 (0.5%)	
Other	1,229 (5.1%)	4,242 (5.2%)	
Payer Mix (Median)			
Medicare	30%	30%	< 0.001
Medicaid	30%	20%	< 0.001
Other	25%	30%	< 0.001
Insurance	25%	40%	< 0.001
Uncompensated	9%	5%	< 0.001
Veterans Affair	5%	5%	< 0.001
Workers Compensation	5%	5%	< 0.001
Self-Pay	5%	5%	< 0.001
Job Satisfaction			
Not Satisfied	4,116 (17.2%)	13,298 (16.6%)	0.015
Satisfied	19,785 (82.8%)	67,041 (83.4%)	
Burnout			
No Symptoms of Burnout	15,305 (64.0%)	54,847 (68.3%)	< 0.001
One or More Symptoms of Burnout	8,597 (36.0%)	25,485 (31.7%)	
Primary Place of Employment Currently Recruiting/Hiring PAs			
No	13,649 (56.7%)	52,236 (64.7%)	< 0.001
Yes	10,408 (43.3%)	28,534 (35.3%)	
Amount of Time PA Positions Remained Unfilled			
1 month	3,336 (32.2%)	11,121 (39.2%)	< 0.001
2 months	1,546 (14.9%)	4,745 (16.7%)	
3 months	1,902 (18.4%)	5,043 (17.8%)	
4 months	459 (4.4%)	1,241 (4.4%)	
5 months	192 (1.9%)	500 (1.8%)	
6 or more months	2,922 (28.2%)	5,725 (20.2%)	
Plan to Leave Principal Clinical PA Position in the Next 12 Months			
No	21,637 (90.2%)	73,809 (91.5%)	< 0.001
Yes	2,344 (9.8%)	6,820 (8.5%)	
^a^PAs not working in HPSA/MUA setting include responses for ‘no’ and ‘not sure’ ^b^Pearson Chi-Square analysis, Independent-Samples Mann-Whitney U Test when appropriate ^c^Primary care (Family medicine, Internal Medicine–General, Pediatrics–General) ^d^FQHC = Federally Qualified Health Center Source: 2022 National Commission on Certification of Physician Assistants (NCCPA) PA Professional Profile			

### Clinical services and payer mix

**Fig. 1:**
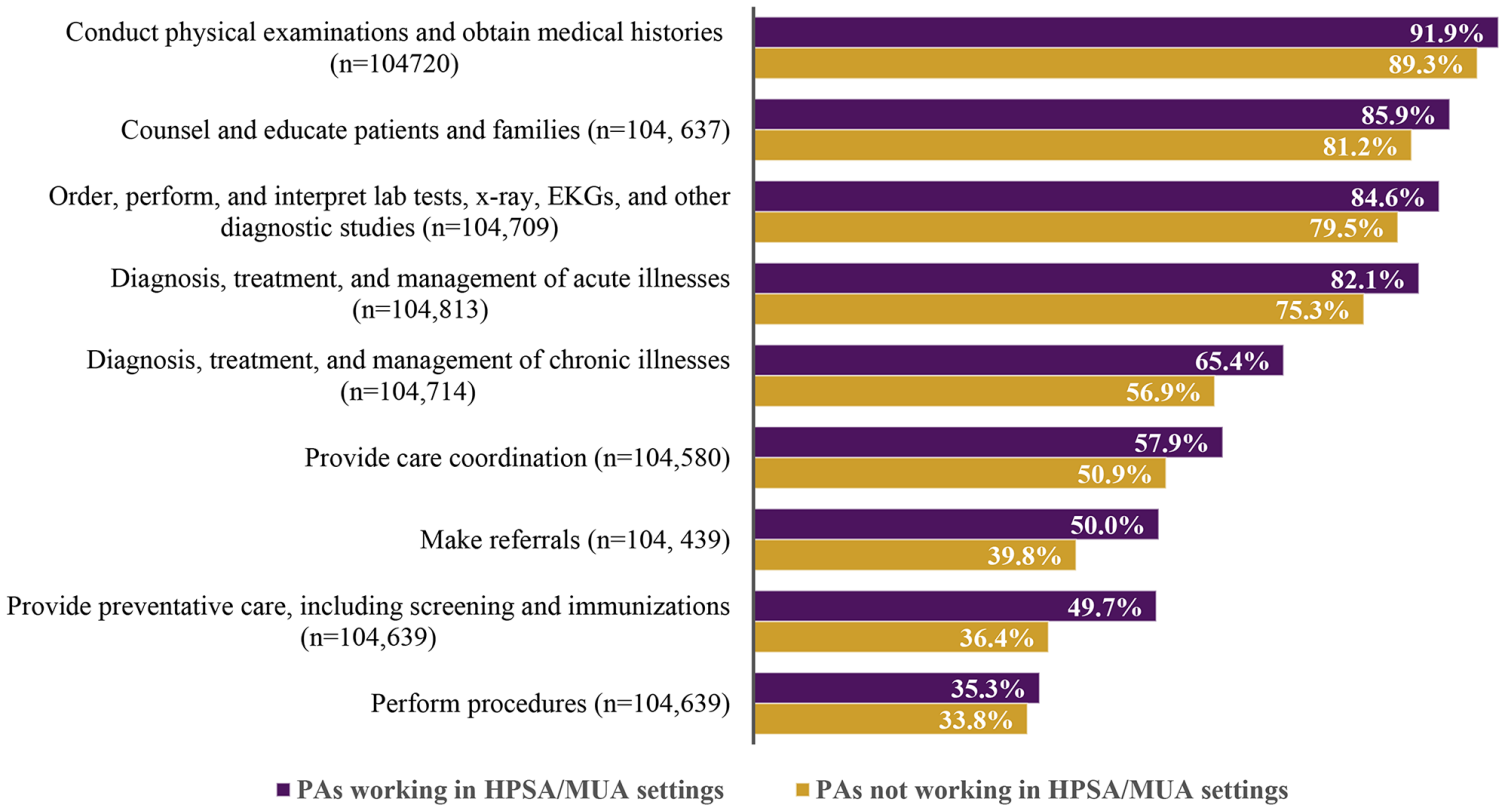
Clinical services provided by PAs working in HPSA/MUA settings vs. non-HPSA/MUA settings for most patients^a^ (*N* = 106,253; all *p* < 0.001^b^) ^a^PAs were asked what proportion of their patients they provide the listed services for in their practice. These percentages are for ‘most’ patients ^b^Pearson Chi-Square analysis Data source: 2022 National Commission on Certification of Physician Assistants (NCCPA) PA Professional Profile

To assess clinical activity, the PA Profile includes a question about what types of services the respondent provides to most of their patients. For each type of service assessed, a higher proportion of PAs working in HPSAs/MUAs than PAs in non-HPSA/MUAs (all *p* < 0.001) reported providing these clinical services to most patients they see (Fig. 1). The greatest differences were found for services typically associated with primary care, including providing counseling (85.9% vs. 81.2%), managing chronic illnesses (65.4% vs. 56.9%), coordinating care (57.9% vs. 50.9%), making referrals (50.0% vs. 39.8%), and providing preventive care (49.7% vs. 36.4%). PAs in HPSAs/MUAs vs. those in other settings also reported that a higher proportion of their services is paid by Medicaid (median of 30% compared to 20%; *p* < 0.001) and uncompensated (median of 9% compared to 5%; *p* < 0.001) (Table 2).

### Job satisfaction

Comparing job satisfaction between PAs working and not working in HPSAs/MUAs revealed that a minimally but statistically significantly lower percentage of those working in HPSAs/MUAs reported job satisfaction (82.8% versus 83.4%; *p* = 0.015). In addition, compared to PAs not working in HPSA/MUA settings, a higher proportion of those in HPSAs/MUAs reported one or more symptoms of burnout (36.0% versus 31.7%; *p* < 0.001) and planned to leave their principal clinical position within the next 12 months (9.8% versus 8.5%; *p* < 0.001) (Table 2).

When examining which factors were considered important in deciding to leave their principal clinical position, five statistically significant differences (all *p* < 0.05) emerged. Among PAs intending to leave their principal clinical position, those practicing in HPSAs/MUAs vs. not were more likely to identify 1) “feelings of professional burnout,” 2) “want to pursue additional education,” 3) “want to work in a health professional training program,” 4) ”work responsibilities would interfere with the ability to care for family” and 5) “other” **(**Appendix A).

### Predictors of practice in HPSA/MUA settings

**Fig. 2:**
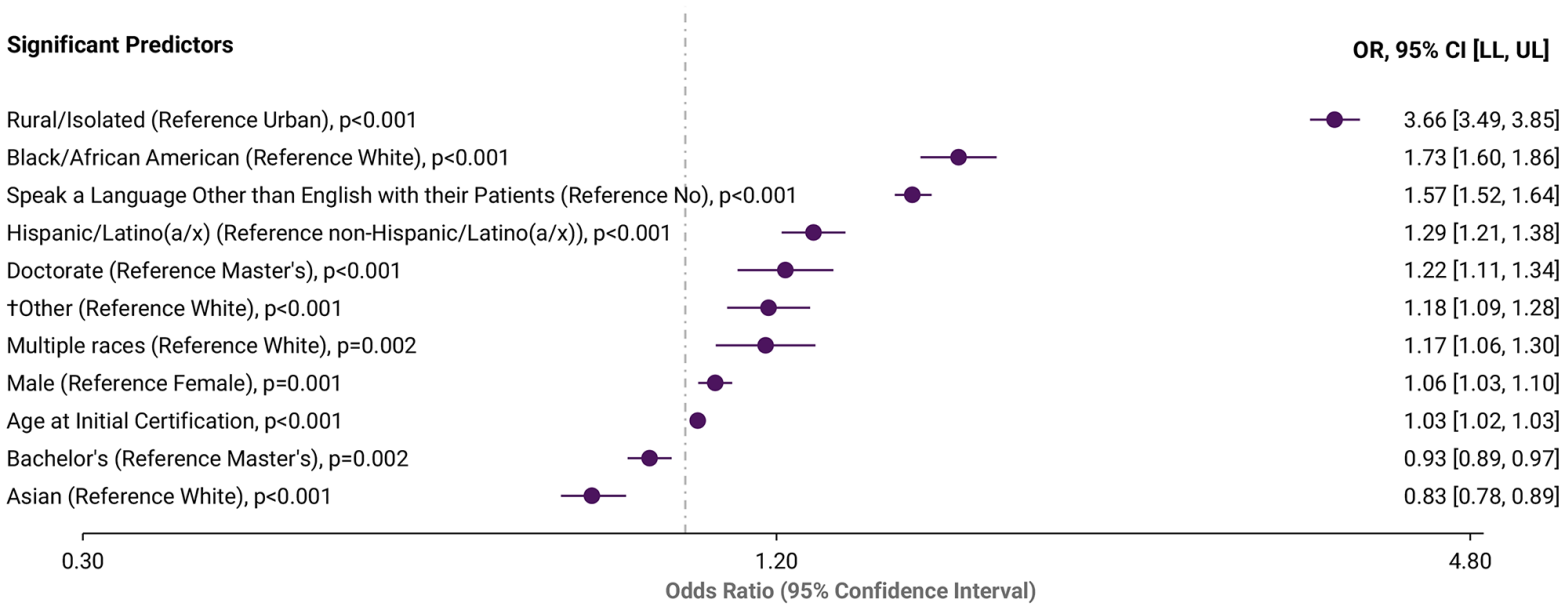
Significant predictors for PAs practicing in HPSA/MUA settings (*N* = 106,253) Adjusted multivariate logistic regression model (Appendix B shows the full regression model) †“Other” includes “other not specified,” “American Indian/Alaska Native”, “Native Hawaiian/Pacific Islander” *Abbreviations*: OR = Odds Ratio; CI = Confidence Interval; LL = Lower Limit; UL = Upper Limit *Source*: 2022 National Commission on Certification of Physician Assistants (NCCPA) PA Professional Profile

Figure 2 displays results of the multivariate logistic regression of potential predictors of practice in a HPSA/MUA setting. Factors associated with higher odds of providing care in a HPSA/MUA setting (all *p* < 0.001) included residing in a rural/isolated location (Odds Ratio [OR] = 3.66, 95% Confidence Interval [95% CI: 3.49, 3.85]), identifying as Black/African American (OR = 1.73, 95% CI [1.60, 1.86]), identifying as Hispanic/Latino(a/x) (OR = 1.29, 95% CI [1.21, 1.38]), male gender (OR = 1.06, 95% CI [1.03,1.10]), older age at initial certification (OR = 1.03, 95% CI [1.02, 1.03]) and speaking a language other than English with patients (OR = 1.57, 95% CI [1.52, 1.64]). While PAs holding a bachelor’s degree (vs. Master’s) (OR = 0.93, 95% CI [0.89, 0.97] and those identifying as Asian (vs. White) (OR = 0.83, 95% CI [0.78, 0.89] had lower odds of working in HPSA/MUA settings. Appendix B displays the full adjusted results of the multivariate logistic regression model.

## Discussion

We found that similar to characteristics of physicians most likely to practice in underresourced communities, PA characteristics associated with greater odds of working in an HPSA/MUA setting included older age at the time of initial certification, male gender, identifying from an URiM group, and speaking a language other than English with patients [29–31]. Unsurprisingly, living in a rural area, which is where the majority of HPSAs/MUAs are located, was associated with more than three times the odds of practicing in a HPSA/MUA. The PA Profile data we analyzed did not include an item to assess geographic location of upbringing. However, prior studies show that growing up in a rural area is among the strongest predictors of rural practice among PAs, as well as physicians and NPs [31, 32].

Interestingly, while less than 3% of PAs possess a doctorate degree [17], our analysis revealed that having a doctorate was also associated with slightly greater odds of practice in a HPSA/MUA. It is possible that having a doctorate signifies pursuit of PA as a second career (i.e., after an initial career requiring a doctorate), suggesting that second-career PAs may be more likely to practice in underresourced areas. Another possibility is that those with advanced training are more confident in their ability or better prepared to manage challenges associated with working in low-resource environments. The finding that older age at the time of initial certification was associated with slightly higher odds of working in a HPSA/MUA supports these notions. However, because PA Profile respondents were not asked whether they obtained their doctorate prior to PA school, this view is speculative.

Over the past decade, the number of clinically active PAs has increased from approximately 90,000 to nearly 169,000 [17]. Yet, despite increasing racial and ethnic diversity in the US population, the proportion of PAs who identify as coming from a racial or ethnic minority background has remained stagnant [17]. As of the end of 2022, just 3.3% and 7.0% of PAs reported their racial and ethnic groups as Black/African American and Hispanic/Latino(a/x), respectively [17]. At the same time, the proportion of PAs who are women has steadily increased over time to 70.6% in 2022 [17]. Our finding that PAs from groups URiM and who identify as male were more likely to work in underresourced areas suggests that any progress toward greater racial and ethnic diversity among PAs or, to a lesser extent, an increase in the proportion of PAs who are men, may help the profession maximize its contributions to care in underresourced communities.

Our study, indicating a higher likelihood of PAs from URiM racial/ethnic groups practicing in underresourced areas, is consistent with earlier research from other healthcare professions. Studies of physicians and other allied health providers have noted that providers from URiM racial/ethnic groups are more likely than providers from other racial/ethnic groups to work in underresourced areas. Rabinowitz and colleagues analyzed national data of 1,704 primary care physicians in 1993 and determined that being from a medically underserved racial/ethnic minority group (Black, Hispanic/Latinx, Native American/Alaska Native), participating in the National Health Service Corps (NHSC), having a strong desire to practice in MUAs before medical school, and growing up in a MUA setting were factors associated with higher likelihood in practicing in MUAs [33]. A later study by Wayne et al. (2010) found similar results: rural background, older age (> 25 years) at matriculation, and URiM racial/ethnic background were predictive of physicians practicing in MUA settings [29]. More recently, Hartman et al. (2022) conducted a multivariate analysis examining the characteristics of doctors of physical therapy (DPT) students and discovered that students who were multilingual, participated in service learning, and grew up in MUA locations had higher odds of practicing in MUAs [34].

Nearly 23% of PAs reported working in HPSAs/MUAs, and among all PAs working in HPSA/MUA settings, more than a third (34.4%) indicated working in primary care. Moreover, a higher percentage of PAs in HPSAs/MUAs reported delivering primary care services, such as preventive care, to most patients. Thus, a substantial portion of the PA workforce appears to be delivering primary care in shortage areas. PAs practicing in HPSA/MUA settings also reported that a higher percentage of their visits were uncompensated or paid by Medicaid, and a lower percentage were paid by private insurance. This is consistent with the fact that individuals under age 65 living in rural areas, as well as those served by FQHCs, are more likely than other residents to be uninsured or have Medicaid [35]. This result also aligns with research showing that residing in a rural area and having a lower income were associated with a greater likelihood of a patient seeing an APP versus a physician [12].

Due to the need for providers in HPSAs/MUAs, retention is a priority. While burnout contributes to provider turnover, research on burnout among clinicians working in underresourced settings is mixed. Studies indicate that high workloads, lack of administrative support, and limited resources are among the factors that contribute to high levels of clinician burnout and job dissatisfaction in underresourced settings [7, 36, 37]. Moreover, clinicians working in FQHCs have a greater likelihood of reporting burnout and intention to leave [38, 39]. Yet, a national study found no difference in burnout among family physicians in rural versus urban areas [40]. Similarly, a large study involving NPs in primary care practices found that while work environment significantly influenced the odds of burnout and job dissatisfaction (better work environment lowered the odds of both), HPSA status did not [7].

We found that a high proportion of PAs practicing in HPSA/MUA settings were satisfied with their jobs (82.8%). However, higher proportions of those in HPSAs/MUAs reported clinic vacancies and PA positions remaining unfilled for six or more months. Higher proportions (36.0%) also reported at least one symptom of burnout and the intention to leave their principal clinical position within the next 12 months. In addition, a slightly higher percentage of PAs in HPSAs/MUAs identified feelings of professional burnout as an important reason for leaving a principal clinical position. While these results are consistent with prior research showing that PAs have high rates of job satisfaction despite relatively high levels of burnout [41], they may suggest that, among PAs, burnout and job turnover are, in fact, more prevalent among those working in HPSA/MUA settings.

Nonetheless, studies involving different types of underresourced settings (e.g., safety-net clinics, rural clinics) suggest that greater control over workload and more administrative support may improve PA retention in underresourced areas [36, 42, 43]. Furthermore, we found that higher percentages of PAs in HPSAs/MUAs indicated that wanting to pursue additional education and wanting to work in a health professional training program were important reasons for leaving a principal clinical position. Thus, creating opportunities for PAs working in HPSAs/MUAs to engage in educational endeavors may also be useful to increase retention.

It should also be noted that while PAs in HPSAs/MUAs were slightly older (at initial certification) than those in other settings, more than half (53.8%) had been certified for less than 10 years, and a minority (20.9%) identified retirement as an important reason for leaving a PA position. Consequently, if PAs that practice in HPSAs/MUAs are retained, they have the potential to make sustained contributions to delivering care to underresourced populations.

## Limitations

Although we analyzed national data, and the item response rate was high (89.7%), because this study is cross-sectional, associations should not be interpreted as causal. In addition, data were self-reported, and many responses were collected during the COVID-19 pandemic, which may have negatively influenced answers to questions about burnout and intent to leave one’s clinical position [44]. Finally, nearly 19.7% of respondents reported that they were ‘not sure’ whether they worked in a HPSA/MUA. Therefore, while the ‘not sure’ option likely minimized the number of respondents who incorrectly reported working in a HPSA/MUA, some may have misidentified their work setting. Our approach did not allow us to confirm the self-reported practice locations (i.e., HPSA/MUAs versus not HPSA/MUAs). Future studies should analyze HRSA data to verify the designation of these locations and validate our findings. Despite these limitations, the study offers potentially valuable insights into the PA workforce providing care in underresourced communities.

## Conclusion

Analyzing a large national dataset, we found that nearly 23% of PAs work in HPSA/MUA settings. Additionally, our findings support and update prior research indicating that those with URiM backgrounds are more likely to practice in underresourced settings and that PAs working in HPSA/MUA versus other environments are more likely to provide primary care services [18–20]. While job satisfaction was high, a higher proportion of PAs working in HPSA/MUA settings reported feelings of professional burnout. As the PA profession grows, knowledge of the characteristics and experiences of those in HPSAs/MUAs may help inform efforts to expand their contributions to address provider shortages.

## Policy implications

With growth in the number of PAs outpacing that of physicians [13], policymakers may increasingly look to PAs to fill gaps in care. Already, PAs manage a higher proportion of visits in rural areas and safety-net clinics than they do in other settings [14, 45]. Based on the characteristics of PAs most likely to work in HPSAs/MUAs, to maximize the numbers who choose to practice in these settings, the PA community must focus efforts on recruiting and admitting individuals from URiM racial/ethnic groups into PA educational programs. In addition, efforts to reduce burnout and promote retention should be tailored to specific challenges faced by PAs as well as other clinicians providing care in underresourced settings.

### Electronic supplementary material

Below is the link to the electronic supplementary material.

Supplementary Material 1

## Data Availability

The datasets generated and analyzed during the current study are not publicly available due to the confidentiality of individualized data, but de-identified data can be available if requested by the corresponding author.

## Abbreviations

HPSA: health professional shortage areas
MUA: medically underserved areas
PA: physician assistant/associate
URiM: underrepresented in medicine
US: United States
HRSA: Health Resources and Services Administration
NP: nurse practitioner
FQHC: federally qualified health centers
NCCPA: The National Commission on Certification of Physician Assistants
CME: continuing medical education
NHSC: National Health Service Corps
DPT: doctor of physical therapy
