# Supplementary material for: Providing care in underresourced areas: contribution of the physician assistant/associate workforce
Source: BMC Health Serv Res. 2024 Jul 25;24:844. doi: 10.1186/s12913-024-11190-x (PMC11282839; doi:10.1186/s12913-024-11190-x)
Supplement: Supplementary file 1 — Supplementary Material 1 [file 12913_2024_11190_MOESM1_ESM.docx]

**Appendix A.** Decision to leave principal clinical PA position of PAs working in HPSA/MUA settings vs. all other settings (N=9,164)

|  | **PAs working in HPSA/MUA settings**  **(N=2,344)** | **PAs not working in HPSA/MUA settings**^a^  **(N=6,820)** | **P-value**^b^ |
| --- | --- | --- | --- |
| **Feelings of professional burnout (N=8,238)** |  |  |  |
| Important^c^ | 1,678 (79.6%) | 4,733  (77.2%) | 0.023 |
| Not important | 430 (20.4%) | 1,397 (22.8%) |  |
| **Want to pursue additional education (N=8,205)** |  |  |  |
| Important | 570 (26.9%) | 1,382 (22.7%) | <0.001 |
| Not important | 1,547 (73.1%) | 4,706  (77.3%) |  |
| **Want to work in a health professional training program (N=8,144)** |  |  |  |
| Important | 444 (21.2%) | 1,041  (17.2%) | <0.001 |
| Not important | 1651 (78.8%) | 5,008  (82.8%) |  |
| **Work responsibilities would interfere with the ability to care for family (N=8,318)** |  |  |  |
| Important | 1,193 (55.9%) | 3,298  (53.3%) | 0.042 |
| Not important | 942 (44.1%) | 2,885  (46.7%) |  |
| **My health does not allow me to continue working as a PA (N=8,121)** |  |  |  |
| Important | 240 (11.5%) | 640  (10.6%) | 0.294 |
| Not important | 1,856 (88.5%) | 5,385  (89.4%) |  |
| **Insufficient wages given the workload and responsibilities (N=8,430)** |  |  |  |
| Important | 1,583 (73.1%) | 4,478  (71.5%) | 0.143 |
| Not important | 582 (26.9%) | 1,787  (28.5%) |  |
| **Work is not professionally challenging or satisfying (N=8,282)** |  |  |  |
| Important | 1,177 (55.2%) | 3,360  (54.7%) | 0.688 |
| Not important | 957 (44.8%) | 2,788  (45.3%) |  |
| **Desire a non-clinical health-related position (e.g., research, administration) (N=8,237)** |  |  |  |
| Important | 491 (23.0%) | 1,476  (24.1%) | 0.367 |
| Not important | 1,629 (76.8%) | 4,641  (75.9%) |  |
| **Desire a position outside of health care (N=8,260)** |  |  |  |
| Important | 488 (23.0%) | 1,488(24.2%) | 0.252 |
| Not important | 1,633 (77.0%) | 4,651 (75.8%) |  |
| **Seeking another clinical PA position (N=8,514)** |  |  |  |
| Important | 1,698 (77.5%) | 4,979 (78.7%) | 0.222 |
| Not important | 493 (22.5%) | 1,344 (21.3%) |  |
| **Relocating to another geographic area (N=8,272)** |  |  |  |
| Important | 950 (44.6%) | 2,612  (42.5%) | 0.087 |
| Not important | 1,178 (55.4%) | 3,532  (57.5%) |  |
| **Plan to retire from the active workforce (N=8,416)** |  |  |  |
| Important | 450 (20.8%) | 1307  (20.9%) | 0.934 |
| Not important | 1,712 (79.2%) | 4,947  (79.1%) |  |
| **Othe**r **(N=5,441)** |  |  |  |
| Important | 444 (32.3%) | 1,191 (29.3%) | 0.036 |
| Not important | 931 (67.7%) | 2,875  (70.7%) |  |

^a^PAs not working in HPSA/MUA setting include responses for ‘no’ and ‘not sure’

**^b^**Pearson Chi-Square analysis.

^c^Important includes ‘very important’ and ‘somewhat important’

Source: 2022 National Commission on Certification of Physician Assistants (NCCPA) PA Professional Profile

**Appendix B.** Multivariate logistic model of PAs practicing in HPSA/MUA settings (N=106,253)

|  |  |  |  |  | 95% CI | |  |
| --- | --- | --- | --- | --- | --- | --- | --- |
|  | **OR** | **S.E.** | **Wald** | **df** | **LL** | **UL** | **P-value** |
| Age at Initial Certification | 1.03 | 0.001 | 315.11 | 1 | 1.02 | 1.03 | <0.001 |
| Speaks a Language Other than English with patients (*Reference No*) | 1.57 | 0.020 | 533.54 | 1 | 1.52 | 1.64 | <0.001 |
| Males (*Reference Females*) | 1.06 | 0.017 | 11.92 | 1 | 1.03 | 1.10 | 0.001 |
| Asian (*Reference White*) | 0.83 | 0.035 | 28.76 | 1 | 0.77 | 0.89 | <0.001 |
| Black/African American (*Reference White*) | 1.73 | 0.039 | 198.46 | 1 | 1.60 | 1.86 | <0.001 |
| Multiple races (*Reference White*) | 1.17 | 0.051 | 9.98 | 1 | 1.06 | 1.30 | 0.002 |
| †Other (*Reference White*) | 1.18 | 0.042 | 15.51 | 1 | 1.09 | 1.28 | <0.001 |
| Hispanic/Latino(a/x) (*Reference non-Hispanic/Latino(a/x)*) | 1.29 | 0.033 | 62.17 | 1 | 1.21 | 1.38 | <0.001 |
| Rural/Isolated *(Reference Urban*) | 3.66 | 0.025 | 2685.21 | 1 | 3.49 | 3.84 | <0.001 |
| Certificate (*Reference Master’s*) | 0.97 | 0.079 | 0.12 | 1 | 0.83 | 1.14 | 0.731 |
| Associate (*Reference Master’s)* | 0.93 | 0.083 | 0.69 | 1 | 0.79 | 1.10 | 0.405 |
| Bachelor’s (*Reference Master’s*) | 0.93 | 0.022 | 10.14 | 1 | 0.89 | 0.97 | 0.001 |
| Doctorate (*Reference Master’s*) | 1.22 | 0.049 | 16.81 | 1 | 1.11 | 1.34 | <0.001 |
| Other (*Reference Master’s*) | 0.92 | 0.100 | 0.61 | 1 | 0.76 | 1.13 | 0.433 |

| Adjusted results of multivariate logistic regression model  †“Other” includes “other not specified,” “American Indian/Alaska Native,” “Native Hawaiian/Pacific Islander” |
| --- |
| Abbreviations: OR=Odds Ratio; CI=Confidence Interval; LL=Lower Limit; UL=Upper Limit; df=Degree of Freedom; S.E.=Standard Error; Wald=Wald test.  Source: 2022 National Commission on Certification of Physician Assistants (NCCPA) PA Professional Profile |
